# Knockdown of *APOPT1/COA8* Causes Cytochrome *c* Oxidase Deficiency, Neuromuscular Impairment, and Reduced Resistance to Oxidative Stress in *Drosophila melanogaster*

**DOI:** 10.3389/fphys.2019.01143

**Published:** 2019-09-06

**Authors:** Michele Brischigliaro, Samantha Corrà, Claudia Tregnago, Erika Fernandez-Vizarra, Massimo Zeviani, Rodolfo Costa, Cristiano De Pittà

**Affiliations:** ^1^Department of Biology, University of Padova, Padua, Italy; ^2^Department of Women and Children’s Health, University of Padova, Padua, Italy; ^3^MRC Mitochondrial Biology Unit, University of Cambridge, Cambridge, United Kingdom; ^4^Department of Neurosciences, University of Padova, Padua, Italy

**Keywords:** *APOPT1*, *Drosophila melanogaster*, cytochrome *c* oxidase deficiency, mitochondrial disease, resistance to oxidative stress, knockdown models

## Abstract

Cytochrome *c* oxidase (COX) deficiency is the biochemical hallmark of several mitochondrial disorders, including subjects affected by mutations in *apoptogenic-1* (*APOPT1*), recently renamed as *COA8* (HGNC:20492). Loss-of-function mutations are responsible for a specific infantile or childhood-onset mitochondrial leukoencephalopathy with a chronic clinical course. Patients deficient in COA8 show specific COX deficiency with distinctive neuroimaging features, i.e., cavitating leukodystrophy. In human cells, COA8 is rapidly degraded by the ubiquitin-proteasome system, but oxidative stress stabilizes the protein, which is then involved in COX assembly, possibly by protecting the complex from oxidative damage. However, its precise function remains unknown. The *CG14806* gene (*dCOA8*) is the *Drosophila melanogaster* ortholog of human *COA8* encoding a highly conserved COA8 protein. We report that *dCOA8* knockdown (KD) flies show locomotor defects, and other signs of neurological impairment, reduced COX enzymatic activity, and reduced lifespan under oxidative stress conditions. Our data indicate that KD of *dCOA8* in *Drosophila* phenocopies several features of the human disease, thus being a suitable model to characterize the molecular function/s of this protein *in vivo* and the pathogenic mechanisms associated with its defects.

## Introduction

Mitochondria are subcellular organelles that play a pivotal role in the conversion of energy stored in nutrients into energy spendable in eukaryotic cells (e.g., ATP and heat). The mitochondrial respiratory chain (MRC) plays a central role in this process. MRC includes four enzymatic multiprotein complexes (named complexes I-IV) embedded in the inner mitochondrial membrane (IMM) that catalyze electron transfer reactions that, through a redox cascade, generate a proton gradient acting as a protonmotive force (PMF) across the IMM. The PMF is then exploited by a fifth complex (complex V, mitochondrial ATP synthase) to convert adenosine diphosphate (ADP) to adenosine triphosphate (ATP), the essential energetic molecule used as a substrate for virtually all endergonic processes in living cells (Spinelli and Haigis, 2018).

Mitochondrial disorders (MD) are a group of mitochondria-related diseases caused by mutations in either nuclear DNA (nDNA) genes, encoding proteins with a role in mitochondrial function, or in mitochondrial DNA (mtDNA) protein or tRNA and rRNA encoding sequences. These diseases are among the most frequently inherited neurometabolic disorders, and are characterized by a wide diversity of clinical features making their diagnosis quite challenging. Thanks to the advances in next generation sequencing (NGS) techniques, the identification of the genetic causes of MD has considerably improved, including extremely rare genetic conditions (Carrol et al., 2014; Legati et al., 2016). For instance, loss-of-function mutations in the *COA8* gene (alias *APOPT1*, *APOP-1*, or *C14ORF153*) have been identified in seven subjects from six different families presenting a distinctive form of mitochondrial encephalopathy (Melchionda et al., 2014; Sharma et al., 2018). The *COA8* mutations include a nonsense mutation (p.Arg79*), a missense mutation (p.Phe118Ser), frameshift mutations (p.Glu121Valfs*4, p.Glu121Valfs*6), and micro-(p.Glu124del) and macro-deletions (p.Val55_Lys120del) (Melchionda et al., 2014; Sharma et al., 2018).

The clinical features include abnormalities of brain magnetic resonance imaging (cavitating leukodystrophy), and neurometabolic failure, including progressive ataxia and spastic tetraparesis (Melchionda et al., 2014; Sharma et al., 2018).

Murine *Apopt1/COA8* was firstly identified as being over-expressed in an *in vitro* model of atherosclerosis (Yasuda et al., 2006). The authors hypothesized a pro-apoptotic role for the protein, because they observed triggering of the apoptotic cascade in a mPTP (mitochondrial permeability transition pore)-dependent manner after its over-expression, causing the release of cytochrome *c* from mitochondria to the cytosol, and the subsequent activation of caspase-9 and caspase-3 (Yasuda et al., 2006; Sun et al., 2008). However, terminal deoxynucleotidyl transferase dUTP nick end labeling (TUNEL) assay failed to detect apoptosis in muscle biopsies from patients, and no difference in growth and apoptotic rate was observed in mutant *versus* control fibroblasts, even after treatment with the apoptosis-inducer staurosporine (Melchionda et al., 2014). In addition, over-expression of a GFP-tagged COA8 protein failed to induce cell death in different human cell lines (Signes et al., 2019), whereas the main biochemical hallmark of its absence was isolated mitochondrial cytochrome *c* oxidase (COX) deficiency (OMIM #220110). Deficiency of COX activity was in fact the only enzymatic abnormality detected in patients’ mitochondria, and was associated in both human and mouse cells and tissues with altered COX assembly (Melchionda et al., 2014; Signes et al., 2019). *COA8* encodes a 206-amino acid protein targeted and localized within mitochondria in mammals (Yasuda et al., 2006; Sun et al., 2008; Melchionda et al., 2014; Signes et al., 2019). Recent evidence proved that it is associated with the inner membrane, with the C-terminal region facing the mitochondrial matrix (Signes et al., 2019).

Analysis of the COX structural defect associated with absence of COA8 revealed its involvement in the intermediate steps of the COX assembly pathway (Signes et al., 2019). In addition, COA8 was shown to be oppositely regulated by UPS and ROS (Melchionda et al., 2014; Signes et al., 2019). However, there is still a need to clarify the regulatory mechanisms and the role of COA8 in COX function as well as in general mitochondrial physiology.

The availability of model organisms can provide valuable insights to clarify the relationship between gene mutations and human diseases. Thus, we have investigated the behavioral and biochemical features of a *D. melanogaster* KD model of *COA8*-associated mitochondrial disease. Our findings indicate that *D. melanogaster* is a suitable model that can significantly contribute to elucidate the role of this gene in mitochondrial physiology and pathology.

## Materials and Methods

### Fly Stocks and Maintenance

Flies were raised on standard cornmeal medium and maintained at 23°C, 70% relative humidity on a 12 h-light and 12 h-dark cycle. The UAS fly strain (w^1118^;P{attP,y+,w3′}VIE-260B; transformant ID 100605) used to perform post-transcriptional silencing, carrying single UAS-*CG14806*-IR autosomal insertion (line ID 100605), was obtained from VDRC (Vienna *Drosophila* Resource Center). The w^1118^ and Gal4 driver lines were obtained from the Bloomington Stock Center (y[1] w[*]; P{w[+mC] = Act5C-GAL4}17bFO1/TM6B,Tb[1] strain ID 3954; P{w[+mW.hs] = GawB}elav[C155] strain ID 458).

### RNA Isolation, Reverse Transcription, and Real-Time Quantitative Reverse Transcription Polymerase Chain Reaction

Total RNA was extracted from 10 adults (whole body) or approximately 30 brains for each genotype (1:1 males-females) using TRIzol reagent (Thermo Fischer Scientific) and miRNeasy Mini Kit (Qiagen) respectively, according to the manufacturer’s instructions. One microgram of total RNA was used for first strand cDNA synthesis employing 10 mM deoxynucleotides, 10 μM oligo-dT and SuperScript II (Life Technologies). Real-time quantitative reverse transcription polymerase chain reactions (qRT-PCRs) were performed in triplicate using a Bio-Rad CFX 96 Touch System (Bio-Rad) using PowerUp SYBR Green chemistry (Thermo Fisher Scientific). The 2^−∆∆*Ct*^ (RQ, relative quantification) method was used to calculate the relative expression ratio (Livak and Schmittgen, 2001). *Rp49* was used as endogenous control and the oligonucleotides employed were *Rp49*-Fw (5′-ATCGGTTACGGATCGAACAA-3′) and *Rp49-*Rv (5′-GACAATCTCCTTGCGCTTCT-3′). The *dCOA8* oligonucleotides used were *dCOA8*-Fw (5′-CAATAAGCGCTTCTACGAGGA-3′) and *dCOA8*-Rv (5′-CCAGTTCTTGTCGAGGAACG-3′).

### Analysis of Spontaneous Locomotor Activity

The amount of locomotor activity was measured by the DAMSystem3 Data Collection Software (Trikinetics). Ten 2-day-old male flies were placed into a glass tube containing food and water in the form of gel at the bottom. Glass tubes were placed into DPM population monitors (Trikinetics) vertically oriented. One day (24 h) after anesthesia, locomotor activity was recorded for 2 days (48 h) under 12:12 light/dark cycles (LD 12:12) at 23°C. At least three biological replicates per genotype were analyzed.

### Climbing Test

Climbing test was performed using a modified version of the countercurrent apparatus originally designed by Seymour Benzer (Benzer, 1967). Twenty 2-day-old flies were placed into the first tube, tapped to the bottom and allowed to climb a 10-cm distance for 10 s. The flies that reached the 10-cm distance were shifted to a second tube, tapped again to the bottom and allowed to climb for further 10 s. The procedure was repeated for a total of five times. At the end, the number of flies in each tube was counted. Climbing indexes were calculated as the weighted average of flies in the different tubes, divided by five times the number of flies in the test. The test was performed 1 h after the dark–light transition and a minimum number of 60 individuals per sex and genotype were analyzed.

### Lifespan Assay and Paraquat Treatment

Flies were reared at standard low density, collected after hatching and divided into males and virgin females over a 24-h window. Adults of the same sex were kept at a density of 10 per vial (for a total of 50 individuals) at 23°C. Flies were counted every day and transferred to fresh medium three times per week, with no anesthesia (Broughton et al., 2005). Lifespan was analyzed both in flies fed with standard food and with food containing 20 mM Paraquat. Standard cornmeal was cooled to 35°C before the addition of Paraquat (methyl viologen dichloride hydrate, Sigma) and then poured into plastic vials.

### Isolation of Mitochondria

Mitochondria were prepared by differential centrifugation from 100 flies (1:1 males-females) as described previously (Da-Rè et al., 2014a). Briefly, samples were homogenized with a Dounce glass potter and a loose-fitting glass pestle in 10 ml of isotonic isolation buffer (225 mM mannitol, 75 mM sucrose, 5 mM HEPES, 1 mM EGTA, pH 7.4) with 1% BSA. Samples were centrifuged at 1,000 ×*g* (Eppendorf 5810R) at 4°C for 10 min. The supernatant was filtered through a fine mesh, and centrifuged at 6,000 ×*g* at 4°C for 10 min. The mitochondrial pellet was washed in 10 ml of isolation buffer and centrifuged at 6,000 ×*g* for 10 min. The wash was repeated using 10 ml of isolation buffer without BSA and centrifuged at 7,000 ×*g* for 10 min. The mitochondrial pellet was resuspended in minimal volume of isolation buffer without BSA. Protein concentration was measured by the Bradford assay (Bio-Rad protein assay).

### Enzymatic Analysis

Prior to enzymatic MRC complex activity assays, isolated mitochondria were subjected to three freeze-thaw cycles in 10 mM ice-cold Tris hypotonic buffer (pH 7.6) using liquid nitrogen to disrupt the mitochondrial membranes. The activities of mitochondrial respiratory chain complexes and citrate synthase (CS) were measured by spectrophotometry as described previously (Kirby et al., 2007), with minor modifications to the protocols.

### Cell Cultures

The *Drosophila* S2R+ cell line is derived from a primary culture of late stage (20–24 h old) *D. melanogaster* embryos (Schneider, 1972) and it was obtained from *Drosophila* Genomics Resource Center (DGRC). S2R+ cells grow at 25°C without CO_2_ in Schneider’s medium (Thermo Fisher Scientific) with 10% heat-inactivated fetal bovine serum (FBS) (Euroclone) as a loose, semi-adherent monolayer, showing a doubling time of about 48 h.

### Cell Transfection and Subcellular Localization

*CG14806* cDNA was cloned in pAc5-STABLE2-neo vector, fused with the EGFP reporter. S2R+ cells were transfected in 24 wells plate using Effectene Transfection Reagent (Qiagen) according to the manufacturer’s instructions. After 48 h, cells were washed once with 1X PBS and incubated with 10 nM MitoTracker Red CMXRos (Thermo Fischer Scientific) and 1 μg/ml cyclosporin H (Sigma) in Schneider’s Medium for 20 min (Da-Rè et al., 2014b). After three washes in PBS, cells were fixed in 4% paraformaldehyde for 20 min. After a final wash in PBS, the slides were mounted with Vectashield mounting medium (Vector Laboratories). Images were taken with a Zeiss LSM700 confocal microscope at 63× magnification.

### Cell Death Analysis

Transiently transfected cells were collected and stained with Annexin V Apoptosis Detection Set PE-Cyanine7 (eBioscience-ThermoFisher Scientific) and propidium iodide (Roche Biochemicals) according to the manufacturer’s protocol. Cells were analyzed using Cytomics FC500 (Beckman Coulter) as described previously (Cusumano et al., 2018).

### Electron Microscopy

Thoraxes and brains from adult male flies were fixed in 2.5% glutaraldehyde overnight. Samples were rinsed in 0.1 M cacodylate buffer with 1% tannic acid and then fixed in 1:1 2% OsO_4_ and 0.2 M cacodylate buffer for 1 h. Samples were rinsed, dehydrated in ethanol, and embedded in Epon. Ultrathin sections (400 Å) were examined and photographed with a FEI Tecnai G2 electron microscope.

### Blue Native Gel Electrophoresis

Isolated mitochondria were resuspended in 1.5 M aminocaproic acid, 50 mM Bis-Tris/HCl pH 7.0. The samples were solubilized with 4 mg digitonin (Merck) per mg of protein. After 5 min. of incubation on ice, samples were centrifuged at 18,000 ×*g* at 4°C for 10 min. The supernatant was collected and resuspended with Sample Buffer (750 mM aminocaproic acid, 50 mM Bis-Tris/HCl pH 7.0, 0.5 mM EDTA, and 5% Serva Blue G). Native samples were run in NativePAGE 3–12% Bis-Tris gels (Thermo Fischer Scientific) according to the manufacturer’s protocol.

### In Gel Activity

For the detection of the activity of mitochondrial respiratory chain complexes, gels were stained with the following solutions:

1. Complex I (NADH:ubiquinone oxidoreductase): NADH: 5 mM Tris–HCl pH 7.4, 0.14 mg/ml NADH (Roche), and 1 mg/ml nitrotetrazolium blue chloride (Sigma);
2. Complex II (succinate dehydrogenase): 5 mM Tris–HCl pH 7.4, 0.2 mM phenazine methosulfate (Sigma), 20 mM succinate, and 1 mg/ml nitrotetrazolium blue chloride;
3. Complex IV (cytochrome *c* oxidase): 50 mM potassium phosphate pH 7.4, 1 mg/ml 3′,3′-diaminobenzidine tetrahydrochloride hydrate (Sigma), 24 units/ml catalase from bovine liver (Sigma), 1 mg/ml cytochrome *c* form equine heart (Sigma), and 75 mg/ml sucrose.

## Results and Discussion

### *CG14806* Is the *Drosophila melanogaster* Ortholog of Human *COA8*

**Figure 1:**
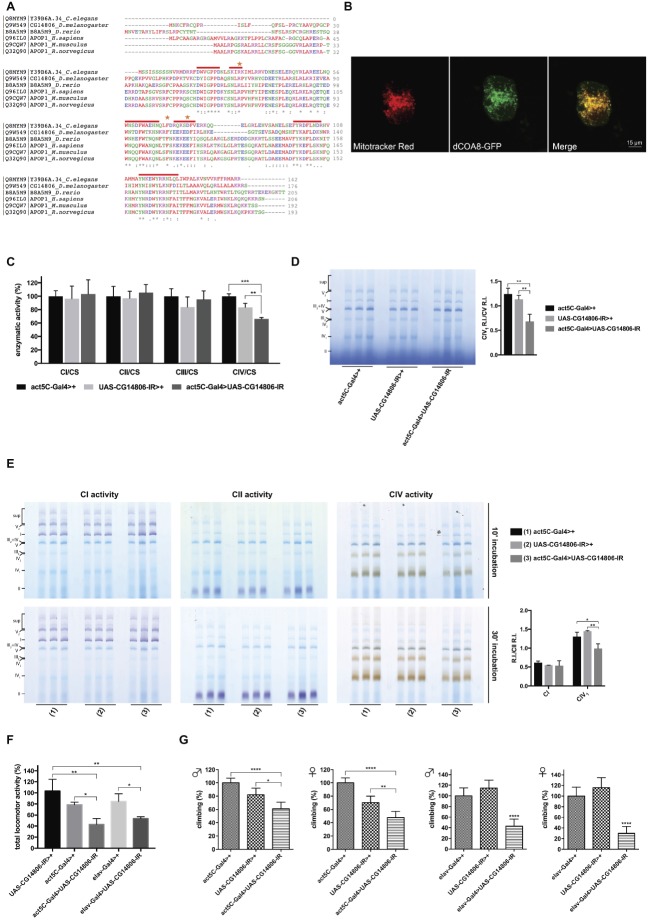
Identification and characterization of the *D. melanogaster* ortholog of *H. sapiens* COA8 (APOPT1). **(A)** Amino acid sequence alignment of *C. elegans*, *D. melanogaster*, *D. rerio*, *H. sapiens*, *M. musculus*, and *R. norvegicus* COA8 orthologs. The shown alignment was performed using the Multiple Sequence Alignment Tool Clustal Omega and it highlights identical residues (*) and similar ones (. and:), conserved patterns and motifs (red lines) and the point mutations found in patients (orange stars). **(B)** Subcellular localization of dCOA8. *Drosophila* cells were transiently transfected with dCOA8-GFP (green), incubated with MitoTracker dye (red), and analyzed by confocal microscopy. Overlay of images (yellow) confirmed the mitochondrial localization of dCOA8. **(C)** Enzymatic activities of MRC complexes (I–IV) were measured in parental controls (act5C-Gal4>+ and UAS-CG14806-IR>+) and in ubiquitous KD flies (act5C-Gal4>UAS-CG14806-IR, dark gray column). Activities of complexes I–IV were normalized to the activity of citrate synthase (CS). For each genotype, three biological replicates of mitochondrial preparations (100 flies for each biological replicate) were analyzed. For each, enzymatic activities from at least 3 replicate reactions were performed. Data plotted are mean ± S.D. (one-way ANOVA with Sidak’s multiple comparisons test *p ≤ 0.05, **p ≤ 0.01, ***p ≤ 0.001). **(D)** 1D-BNGE analysis of MRC complexes and quantification of the relative intensity (RI) of CIV_1_ bands normalized to the R.I. of CV band of the same sample (one-way ANOVA with Sidak’s multiple comparisons test **p ≤ 0.01). Isolated mitochondria from flies of the indicated genotypes were solubilized and ran in native conditions. **(E)** In gel activity of complex I, complex II, and complex IV after short (10 min) and long (30 min) incubation times and quantification of the relative intensity (RI) of CI and CIV_1_ bands normalized to the R.I. of CII band of the same sample after 10 min of reaction (one-way ANOVA with Sidak’s multiple comparisons test *p ≤ 0.05, **p ≤ 0.01). Genotypes are [(1) act5C-Gal4>+, (2) UAS-CG14806-IR, (3) act5C-Gal4>UAS-CG14806-IR]. **(F)** Spontaneous locomotor activity was measured in ubiquitous (act5C-Gal4>UAS-CG14806-IR) and pan-neuronal (elav-Gal4 > UAS-CG14806-IR) *dCOA8* KD flies with respect to parental controls (UAS-CG14806-IR > +, act5C-Gal4>+, and elav-Gal4>+) for 2 days (48 h). Data plotted are mean ± S.D. (one-way ANOVA with Sidak’s multiple comparisons test *p ≤ 0.05, **p ≤ 0.01). **(G)** The climbing assay was performed in ubiquitous (act5C-Gal4 > UAS-CG14806-IR) and pan-neuronal (elav-Gal4 > UAS-CG14806-IR) *dCOA8* KD flies with respect to parental controls (UAS-CG14806-IR>+, act5C-Gal4>+, and elav-Gal4>+). Charts show mean and 95% CI, *n* = 60 animals. Statistical analysis used one-way ANOVA with Dunn’s multiple comparisons test (*p ≤ 0.05, **p ≤ 0.01, ***p ≤ 0.001, ****p ≤ 0.0001).

*COA8* (*APOPT1*) is present in higher eukaryotes such as *Mus musculus, Rattus norvegicus, Danio rerio, Drosophila melanogaster*, and *Caenorhabditis elegans* but it is absent in lower eukaryotes such as *Saccharomyces cerevisiae.* A *COA8* ortholog is present in the *Drosophila* genome on the X chromosome (*CG14806*, hereafter named *dCOA8*). Two different transcripts of the *dCOA8* gene, both encoding the same 176-amino acid protein, are present in flies. Clustal Omega (Sievers et al., 2011) alignment of the *Drosophila* protein showed 37% of sequence similarity with the human one. Interestingly, human *dCOA8* pathological mutations involve amino acids that are conserved in *Drosophila* (Figure 1A).

COA8 has been shown to localize to mitochondria in both human and murine models (Yasuda et al., 2006; Melchionda et al., 2014) being associated with the inner mitochondrial membrane (IMM), with its C-terminal region facing the matrix (Signes et al., 2019). Likewise, dCOA8 has a predicted N-terminal mitochondrial targeting sequence according to the online prediction tools Target P (Emanuelsson et al., 2000) and MitoProt II (Claros and Vincens, 1996), with a probability score of 0.67 and 0.91, respectively. Notably, there is high inter-species variability in the N-terminal sequences (Figure 1A). Furthermore, the topology prediction tools Phobius (Käll et al., 2004) and TMpred (Hofmann and Stoffel, 1993) indicate the presence of one C-terminal transmembrane domain suggesting that dCOA8 could be associated with a mitochondrial membrane, most likely the IMM due to the presence of an MTS, while the rest of the hydrophilic domains could be localized in the mitochondrial matrix.

We transiently expressed a recombinant form of *dCOA8* fused at the C-terminus with the GFP reporter (*dCOA8-GFP*) in S2R+ cells in order to define the subcellular localization of dCOA8. Colocalization of MitoTracker staining and the GFP signal clearly indicated dCOA8 targeting to mitochondria (Figure 1B).

### *dCOA8* Deficiency Phenocopies Some Human Clinical and Biochemical Features in Flies

*dCOA8* is mainly expressed in the adult brain and thoracic-abdominal ganglion, according to FlyAtlas (Leader et al., 2018). dCOA8 is likely to play an important role in the central nervous system (CNS), in line with the severe neurological manifestations described in the COA8-related disease. To corroborate this, *dCOA8* expression was modulated in a spatial and temporal way by exploiting the UAS-Gal4 system. *dCOA8* was either ubiquitously downregulated, through the act5C-Gal4 driver, or specifically downregulated in the nervous system using the elav-Gal4 driver. *dCOA8* mRNA levels dropped around 60–90% of the control levels in the ubiquitous (whole body) and pan-neuronal (brain) KD flies, respectively (Supplementary Figure S1A). Despite highly efficient silencing, *dCOA8* KD individuals reached the adult stage and showed similar lifespan to the controls (Supplementary Figure S1B). However, kinetic analysis of mitochondrial respiratory chain (MRC) complexes revealed a specific and marked reduction in COX activity in ubiquitous KD flies (Figure 1C). To further explore the COX deficiency and, more generally, MRC complexes activity/amount, isolated mitochondria were subjected to one dimension (1D) blue native gel electrophoresis (1D-BNGE) and the natively separated samples were analyzed by in gel activity (IGA) for complex I, complex II, and complex IV. While the activity of complexes I, II, III, and V were unaffected, the activity of complex IV was clearly reduced in mitochondria from KD flies (Figure 1D). Additionally, the COX-specific IGA reactivity was significantly lower in the dCOA8 KD samples, in the monomeric (IV_1_), dimeric (IV_2_), and super-assembled species (III_2_ + IV and upper bands). Contrariwise, CI and CII activities were comparable to those of parental control mitochondria (Figure 1E).

In order to investigate possible phenotypic consequences of dCOA8 deficiency, we tested the amount of spontaneous locomotor activity and performed a climbing assay, since impairment of these tests would suggest neurological impairment possibly due to neurodegeneration. Both ubiquitous and pan-neuronal *dCOA8* KD flies showed behavioral alterations, with a significant decrease in both total locomotor activity (Figure 1F) and climbing ability (Figure 1G). We also analyzed mitochondrial morphology in thoracic muscles (from ubiquitous KD flies) and brains (from pan-neuronal KD flies) by transmission electron microscopy (TEM), but found no mitochondrial morphological alterations in either tissue (Supplementary Figure S1C).

### dCOA8 Does Not Have a Direct Role in the Apoptotic Process

**Figure 2:**
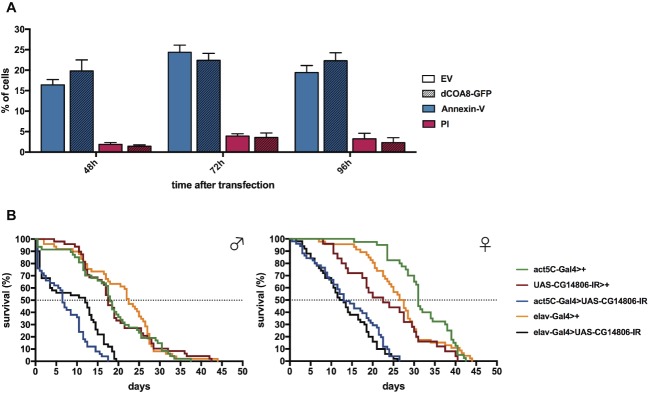
Functional characterization of *dCOA8*. **(A)** Cell death analysis. dCOA8-GFP over-expressing and control S2R+ cells (transfected with an empty vector expressing a cytosolic GFP) were double-stained with Annexin V and propidium iodide (PI). Percentage of early apoptotic (blue bars) and necrotic (red bars) cells was measured 48, 72, and 96 h post-transfection. Data plotted are mean ± S.D. (*n* = 3 biological replicates, two-way ANOVA with Sidak’s multiple comparisons test not significant). **(B)** Representative lifespan curves (Kaplan-Meier) of ubiquitous (act5C-Gal4 > UAS-CG14806-IR) and pan-neuronal (elav-Gal4 > UAS-CG14806-IR) *dCOA8* KD flies with respect to parental controls (UAS-CG14806-IR>+, act5C-Gal4>+, and elav-Gal4>+) under oxidative stress at 23°C. Flies were treated with 20 mM Paraquat in standard food. Statistical analysis was performed with log-rank (Mantel-Cox). All the comparisons between the two KD lines with respect to parental controls are statistically significant (****p ≤ 0.0001).

The possible involvement of dCOA8 in apoptosis was tested using a combination of an Annexin V Apoptosis Detection assay (detecting early apoptotic cells) and a propidium iodide staining (to quantify necrotic cells). Labeled cells were analyzed by cytofluorimetry in *dCOA8*-GFP over-expressing S2R+ cells at very high levels (Supplementary Figure S1D). No difference in the amount of apoptotic or necrotic cells was observed between dCOA8-GFP over-expressing and control cells transfected with an empty vector, carrying a cytosolic GFP (Figure 2A). Therefore, also in *D. melanogaster*, involvement of COA8 in apoptosis failed to be demonstrated.

### *dCOA8* Protects Flies From Oxidative Stress

Previous work on human cellular models showed that COA8 is rapidly degraded by the ubiquitin-proteasome system (UPS) but it is strongly stabilized after treatment with oxidants (Melchionda et al., 2014; Signes et al., 2019). Moreover, COX was especially sensitive to oxidative stress in the absence of COA8, suggesting a specific predominant role of dCOA8 to protect nascent COX under oxidative stress conditions (Signes et al., 2019).

To test the direct involvement of dCOA8 in the reactive oxygen species (ROS) response, *dCOA8* KD flies were treated with Paraquat (20 mM), which catalyzes the formation of superoxide (Farrington et al., 1973). Both ubiquitous and pan-neuronal KD flies showed significant reduction of their lifespan after Paraquat treatment, with median survival rates between 36 and 68% of the parental controls for males, and between 41 and 57% for females (Figure 2B). These data indicate that dCOA8 has a protective role against oxidative stress response *in vivo*, confirming in a living whole organism that this protein is functionally related to ROS response, as previously reported in mammalian cells (Melchionda et al., 2014; Signes et al., 2019).

In conclusion, we generated and characterized *D. melanogaster* models of the human mitochondrial disorder caused by pathogenic mutations in *COA8*. This study shows that *D. melanogaster* is a suitable, user-friendly model to shed light on the molecular and physiological roles of COA8. Further investigation is needed to understand its mechanistic and homeostatic role in COX biogenesis. Ubiquitous and pan-neuronal *dCOA8* knockdown flies showed behavioral and biochemical alterations resembling the clinical features of patients and KO mice, thus demonstrating a phylogenetically essential role in MRC function of higher eukaryotes. *dCOA8* deficiency induces MRC dysfunction with marked reduction in the activity of COX. The link between the over-expression of dCOA8 and the apoptotic process originally proposed in human and mouse models (Yasuda et al., 2006; Sun et al., 2008) was also not confirmed in flies. Interestingly, reduced ability to cope with oxidative challenges was observed in *dCOA8* KD flies, demonstrating for the first time a relevant role of dCOA8 in response to oxidative stress *in vivo*. Thus, our data support the idea that COA8 plays an essential role in protecting nascent COX from oxidative damage particularly during the metallation of the COX catalytic sites. Future work is warranted to clarify this possible function of COA8 in higher eukaryotes.

## Data Availability

The datasets generated for this study can be found in the FlyBase (CG14806).

## Author Contributions

CP and RC conceived and designed the research. MB and SC performed the experiments. MB, SC, and CT analyzed the data. MB, SC, CT, RC, MZ, EF-V, and CP interpreted the results of experiments. MB and CP wrote the manuscript. EF-V, RC, and MZ revised the manuscript.

### Conflict of Interest Statement

The authors declare that the research was conducted in the absence of any commercial or financial relationships that could be construed as a potential conflict of interest.

## Supplementary Material

SUPPLEMENTARY Figure S1 **(A)** *dCOA8* mRNA levels, expressed as relative quantity of template in the sample (RQ), in ubiquitous (act5C-Gal4>UAS-CG14806-IR) and pan-neuronal (elav-Gal4>UAS-CG14806-IR) knockdown flies with respect to parental controls (UAS-CG14806-IR>+, act5C-Gal4>+, and elav-Gal4>+). Data plotted are mean ± S.D. (*n* = 3, Student’s *t* test **p* ≤ 0.05, ***p* ≤ 0.01, ****p* ≤ 0.001, *****p* ≤ 0.0001). **(B)** Lifespan of ubiquitous male (left) and female (right) KD flies in standard food at 23°C. Statistical significance was assessed with log-rank (Mantel-Cox) and Gehan-Breslow-Wilcoxon tests (non-significant). **(C)** Electron microscopy analysis on thoracic muscle (upper panels) and brain (lower panels) sections. Characterization was carried out on ubiquitous (act5C-Gal4 > UAS-CG14806-IR) *dCOA8* KD flies and parental controls (UAS-CG14806-IR>+ and act5C-Gal4>+). Cross-sectional ultrastructure of thoracic muscles and brains, illustrating the distribution and morphology of mitochondria, are represented. **(D)** *dCOA8* mRNA levels, expressed as relative quantity of template in the sample (RQ), in *dCOA8* over-expressing S2R+ (dCOA8-GFP) and control cells (transfected with the empty vector, EV) 24 and 48 h post-transfection. Data plotted are mean ± S.D. (*n* = 3, Student’s *t* test **p* ≤ 0.05, ****p* ≤ 0.001).
