# Supplementary figures and images for: Knockdown of APOPT1/COA8 Causes Cytochrome c Oxidase Deficiency, Neuromuscular Impairment, and Reduced Resistance to Oxidative Stress in Drosophila melanogaster
Source: Front Physiol. 2019 Sep 6;10:1143. doi: 10.3389/fphys.2019.01143 (PMC6742693; doi:10.3389/fphys.2019.01143)

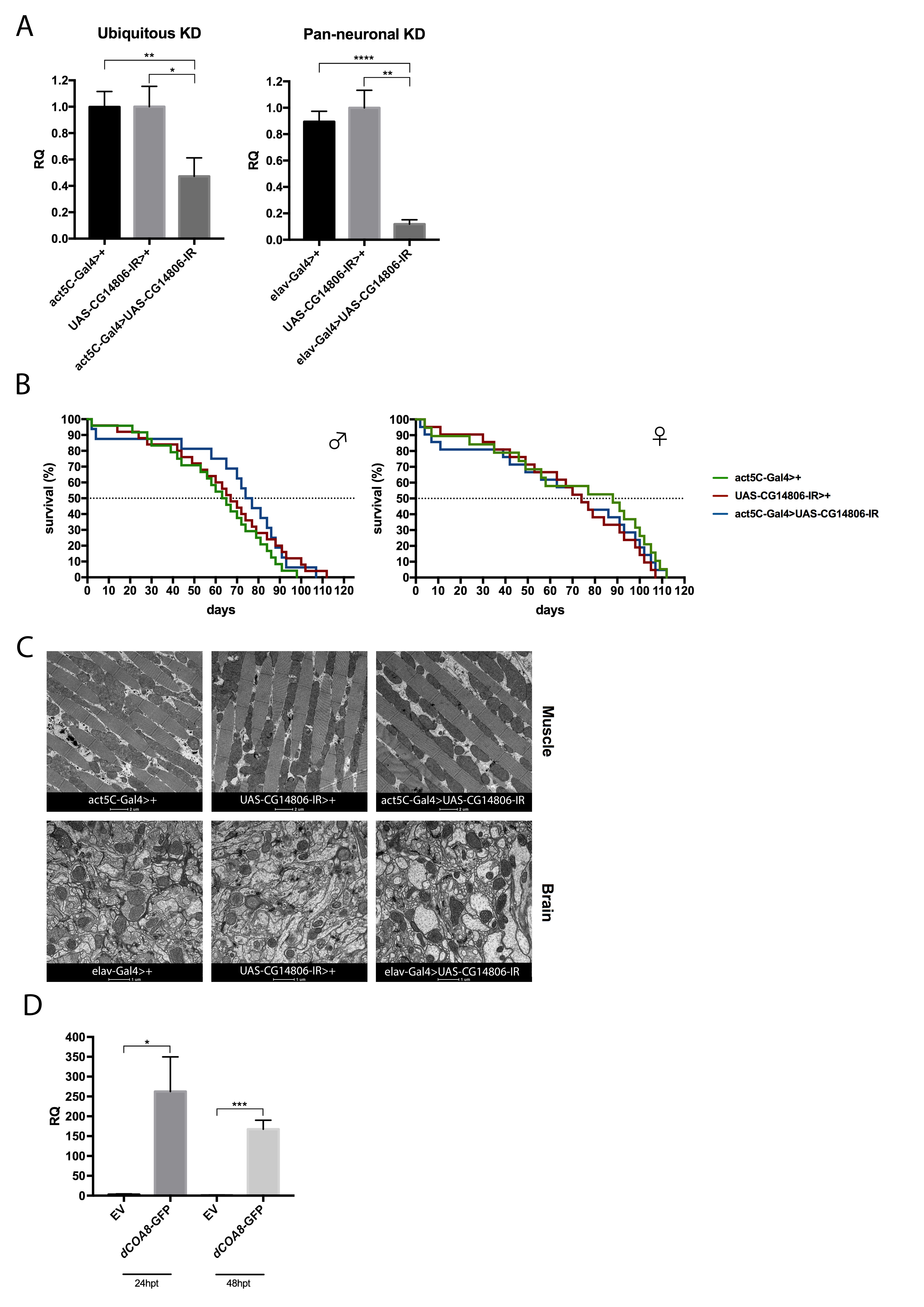

Supplement: SUPPLEMENTARY Figure S1 — (A) dCOA8 mRNA levels, expressed as relative quantity of template in the sample (RQ), in ubiquitous (act5C-Gal4>UAS-CG14806-IR) and pan-neuronal (elav-Gal4>UAS-CG14806-IR) knockdown flies with respect to parental controls (UAS-CG14806-IR>+, act5C-Gal4>+, and elav-Gal4>+). Data plotted are mean ± S.D. (n = 3, Student’s t test *p ≤ 0.05, **p ≤ 0.01, ***p ≤ 0.001, ****p ≤ 0.0001). (B) Lifespan of ubiquitous male (left) and female (right) KD flies in standard food at 23°C. Statistical significance was assessed with log-rank (Mantel-Cox) and Gehan-Breslow-Wilcoxon tests (non-significant). (C) Electron microscopy analysis on thoracic muscle (upper panels) and brain (lower panels) sections. Characterization was carried out on ubiquitous (act5C-Gal4 > UAS-CG14806-IR) dCOA8 KD flies and parental controls (UAS-CG14806-IR>+ and act5C-Gal4>+). Cross-sectional ultrastructure of thoracic muscles and brains, illustrating the distribution and morphology of mitochondria, are represented. (D) dCOA8 mRNA levels, expressed as relative quantity of template in the sample (RQ), in dCOA8 over-expressing S2R+ (dCOA8-GFP) and control cells (transfected with the empty vector, EV) 24 and 48 h post-transfection. Data plotted are mean ± S.D. (n = 3, Student’s t test *p ≤ 0.05, ***p ≤ 0.001). [file Image_1.jpg]
